# Supplementary material for: Underlying medical conditions and anti-SARS-CoV-2 spike IgG antibody titers after two doses of BNT162b2 vaccination: A cross-sectional study
Source: PLoS One. 2023 Apr 6;18(4):e0283658. doi: 10.1371/journal.pone.0283658 (PMC10079051; doi:10.1371/journal.pone.0283658)
Supplement: Attachment — Submitted filename: Response_to_Reviewers.docx [file pone.0283658.s001.docx]

**Manuscript ID: PONE-D-22-29732**

**Underlying medical conditions and anti-SARS-CoV-2 spike IgG antibody titers after two doses of BNT162b2 vaccination: a cross-sectional study**

***PLOS One***

Many thanks for your thoughtful reviews and positive evaluation for our study. We have revised our manuscript in response to your suggestions, and used English editing services to improve the manuscript English quality. Our responses are described in normal font following the reviewers’ comments in boldface. All modifications in the current version of manuscript are highlighted in a font color of red. We hope that our revision will be satisfactory for your publication criteria.

**Reviewer #1:**

**If you don’t mind I have some comments before acceptation, please correct as much as you can:
1. Please start by a more general statement in the first sentence of the background in your abstract.**

Response:

Thank you very much for your insightful comments. We have added a general statement in the abstract.

Lines 1 to 5 in the Abstract.

“Patients with underlying medical conditions are at high risk of developing serious symptoms of the coronavirus disease 2019 than healthy individuals, so that it is necessary to evaluate the immune response to vaccination among them to formulate precision and personalized vaccination strategies. However, inconsistent evidence exists regarding whether patients with underlying medical conditions have lower anti-SARS-CoV-2 spike IgG antibody titers.”

**2. I think you don’t have to specify (95% confidence interval, CI)**

Response:

We have discussed your suggestion, but we think that 95% confidence interval is necessary to show the interval of the true population mean at a 5% level (p < 0.05). We believe it could provide effective information for readers to understand the results. For reference, the following Covid antibodies-related manuscript showed the 95% confidence interval of ratio of mean.

DOI: [10.1056/NEJMoa2114583](https://doi.org/10.1056/nejmoa2114583)

**3. Line 4-5 Please update data**

Response:

We have updated the data on the epidemic status of COVID-19.

Line 3 to 5, page 5.

“Since the first official case was reported, the disease has affected over 644 million and caused the death of 6.6 million individuals worldwide up until December 2022 [2].”

**4. From line 1-7, there is a lack of references in the first part of your introduction, please cite more papers, you don’t have to limit yourself only to papers concerning your country, here are some papers to add:
1.https://doi.org/10.1111/cbdd.13761
2.https://doi.org/10.3390/healthcare10071341
3.https://doi.org/10.1186/s12941-021-00438-7**

Response:

We have added more related studies as references in the introduction, as suggested.

**5. There are also some very important studies concerning vaccination, its side effects, comparison between them and also booster doses, please add these two papers:
1. https://doi.org/10.3390/vaccines10111781
2.** [**https://doi.org/10.3389/fpubh.2022.896343**](https://doi.org/10.3389/fpubh.2022.896343)

Response:

These papers are about inactivated-virus and adenoviral-vector vaccines, while participants received messenger RNA vaccine in our study. Also, we did not discuss mild side effects (pain, fatigue, fever, and etc.) of vaccination, our concern is the difference of IgG antibody titers in different population. So, we did not add these studies as additional references.

**6. Please try to add at least 1-2 sentences in your introduction before your objective.**

Response:

We have modified the description of the purpose of this study for clarity in Introduction.

Line 17 to 26, page 5 to 6.

“Vaccination is a major measure employed to prevent the transmission of coronavirus and contain the COVID-19 pandemic. It is important to evaluate the immune response to vaccination among high-risk groups such as patients with underlying medical conditions to establish precision and personalized vaccination strategies. Given that previous inconsistent findings may be due to small sample size or single center study design, data from a multicenter large-scale population sample could provide critical evidence for evaluating the immune response to vaccination in patients with underlying medical conditions. Therefore, we aimed to investigate the association between underlying medical conditions and anti-SARS-CoV-2 spike IgG antibody titers in healthcare workers from national centers for advanced medical and research in Japan.”

**7. I think it’s better to add a conclusion section instead of including it in the discussion section.**

Response:

We have added a conclusion section as you suggested.

**8. Please put the first lines of your tables in bold**

Response:

We have put in bold the title of all tables.

**9. My last comment is concerning English language, even if the paper is pleasant to read (specifically methods and discussion). However, there are some sentences that doesn’t read well. Please consider revising carefully your paper by a native English, a specialist or an English editing service.**
Response:

We have used an English editing service to improve the English quality in the current version of our manuscript.
